# Running from Disease: Molecular Mechanisms Associating Dopamine and Leptin Signaling in the Brain with Physical Inactivity, Obesity, and Type 2 Diabetes

**DOI:** 10.3389/fendo.2017.00109

**Published:** 2017-05-23

**Authors:** Gregory N. Ruegsegger, Frank W. Booth

**Affiliations:** ^1^Department of Biomedical Sciences, University of Missouri, Columbia, MO, United States; ^2^Department of Medical Pharmacology and Physiology, University of Missouri, Columbia, MO, United States; ^3^Department of Nutrition and Exercise Physiology, University of Missouri, Columbia, MO, United States; ^4^Dalton Cardiovascular Research Center, University of Missouri, Columbia, MO, United States

**Keywords:** physical activity, physical inactivity, motivation, dopamine, obesity, leptin

## Abstract

Physical inactivity is a primary contributor to diseases such as obesity, cardiovascular disease, and type 2 diabetes. Accelerometry data suggest that a majority of US adults fail to perform substantial levels of physical activity needed to improve health. Thus, understanding the molecular factors that stimulate physical activity, and physical inactivity, is imperative for the development of strategies to reduce sedentary behavior and in turn prevent chronic disease. Despite many of the well-known health benefits of physical activity being described, little is known about genetic and biological factors that may influence this complex behavior. The mesolimbic dopamine system regulates motivating and rewarding behavior as well as motor movement. Here, we present data supporting the hypothesis that obesity may mechanistically lower voluntary physical activity levels *via* dopamine dysregulation. In doing so, we review data that suggest mesolimbic dopamine activity is a strong contributor to voluntary physical activity behavior. We also summarize findings suggesting that obesity leads to central dopaminergic dysfunction, which in turn contributes to reductions in physical activity that often accompany obesity. Additionally, we highlight examples in which central leptin activity influences physical activity levels in a dopamine-dependent manner. Future elucidation of these mechanisms will help support strategies to increase physical activity levels in obese patients and prevent diseases caused by physical inactivity.

## Introduction

Physical inactivity presents a major public health problem. Predictions by Lee et al. (1) estimated that physical inactivity accounts for between 6 and 10% of type 2 diabetes (T2D) and coronary heart disease prevalence, with this percentage further elevated for specific diseases (30% for ischemic heart disease) (2). Moreover, the World Health Organization declared physical inactivity as the fourth leading risk factor for death worldwide, responsible for ~6% of the deaths worldwide in 2008 (1, 2). Accelerometry measurements by Troiano et al. (3) reported that less than 5% of adults met the US guidelines for physical activity, while questionnaire data collected globally in 2009 suggested that 31% of the world’s population did not attain minimum recommended levels of physical activity (4). Given the deleterious effects of physical inactivity, understanding molecular mechanisms that influence physical activity adherence is needed. Here, we summarize current knowledge suggesting the mesolimbic dopamine system regulates physical activity, obesity-induced impairments in dopamine signaling may cause physical inactivity, and central leptin resistance in obesity and T2D may alter physical activity in a dopamine-dependent manner. Specifically, our discussion focuses on motivated and self-rewarding (i.e., voluntary wheel running), rather than spontaneous (i.e., cage activity, tremors, etc.), forms of physical activity.

## Genetic Control of Physical Activity

In 1953, Mayer, a leader who helped clarify the natures of hunger and of obesity, demonstrated that physical activity behavior has a biological basis (5). Mayer noted that obese, hyperglycemic mice were far less active than non-obese littermates. However, when the obese mice were bred against mice with a so-called “waltzing gene” physical activity increased to sufficiently prevent the development of obesity. Since Mayer’s original speculation of an uncharacterized “waltzing gene,” studies in animals and humans have estimated the genetic component for physical inactivity to be between 20 and 80% (6–12). Analysis of 772 same-sex twin pairs concluded that 31% of the variance in daily sedentary time was explained by heritable factors (13). Of these heritable factors, associations between dopamine and motivated physical activity are well established, as discussed below. However, other neuromodulators such as endocannabinoids (14, 15), opioids (16), and brain-derived neurotrophic factor (17) also influence physical activity behavior. Furthermore, interactions between these neuromodulatory systems imply that biological networks control voluntary physical activity (18). Evolutionary perspectives also argue that while selection did not operate to cope with the detrimental effects of long-term physical inactivity, humans adapted to avoid unnecessary exertion due to limited energy supply (19). Additionally, gene–environment interactions influence physical activity. Rowland (20) proposed that through components related to energy balance control an “activity-stat” may regulate the propensity for physical activity. Furthermore, obesity was speculated to be a critical negative influencer of the “activity-stat” (21). Collectively, these findings suggest that physical activity levels have strong genetic control.

## Dopaminergic Control of Physical Activity

Although detailed mechanisms describing the neurobiology of wheel running are incomplete, substantial evidence suggests that the mesolimbic dopamine pathway, specifically the ventral striatum and nucleus accumbens (NAc), plays an important role in determining voluntary running behavior (22–24). A detailed review of the mesolimbic dopamine system is beyond the scope of this review; however, a brief overview is provided next [please see Ref. (25, 26) for more detailed review]. In the mesolimbic dopamine system, dopaminergic neurons originating in the ventral tegmental area (VTA) project to various limbic nuclei, including the NAc, and changes in dopamine transmission play central roles in modulating information flow through the limbic system (27–30). These nuclei, through interconnections *via* dopaminergic neurons, have implications in reward, motivation, learning, and motor movement (31). Importantly, the NAc acts as a “filter” and/or “amplifier” of information passing between various limbic, cortical, and motor areas of the brain, suggesting the NAc is instrumental in orchestrating behavioral processes related to motivation (25). Several reports have demonstrated that other mesolimbic structures, such as the VTA and prefrontal cortex, contribute to reward derived from physical activity, potentially through their interactions with the NAc (32–34).

Disruption of dopaminergic transmission and/or dopamine receptor expression in the NAc and ventral striatum can strongly influence voluntary physical activity. The depletion of NAc dopamine by 6-hydroxydopamine decreased wheel running ~40% (35). Knab et al. (22) suggested that differences in dopamine 1-like (D1-like) receptors and tyrosine hydroxylase (*Th*) mRNA, the rate-limiting enzyme in dopamine synthesis, in the NAc influence different running distances between mouse strains.

Selective breeding studies have provided ample insight into voluntary physical activity regulation. Mice bred by Garland et al for high voluntary running distance displayed dysfunctional dopaminergic profiles in the NAc (36, 37) and increased dopamine receptor 2 (*Drd2*) and dopamine receptor 4 (*Drd4*) mRNA ~20% in the hippocampus (38), compared to control mice. Furthermore, agonism (24) and antagonism (37) of D1-like receptors in the NAc paradoxically both decreased wheel running in high-running mice to a greater extent than in control mice. Similar findings from our group using rat lines selectively bred for high (HVR) and low (LVR) wheel-running suggested rats predisposed to run high nightly distances may quickly develop a rewarding response to exercise due to optimal D1-like receptor signaling in the NAc (39). Collectively, these data suggest the following: (1) dopamine signaling is optimally primed to achieve reward associated with running in high-running rats, (2) dopamine is at least partially required for wheel-running behavior, and (3) animals run to achieve the rewarding effects of dopamine but do not want to run when dopamine signaling is artificially activated. Dopamine receptors 1 (*Drd1*), *Drd2*, and dopamine receptor 5 (*Drd5*) mRNA were also inherently 50 to 85% higher in the NAc of HVR compared to LVR (16). Similarly, inherent ~1.3-fold increases in NAc *Drd1* mRNA and ~1.8-fold greater dopaminergic activity were speculated to mediate increased wheel running in rats selectively bred for high, compared to low, aerobic capacity, suggesting that aerobic capacity may influence physical activity levels through alterations in mesolimbic dopamine activity (40, 41). Furthermore, the loss of dopamine receptors or reduced dopamine release in the brain was associated with age-related declines in physical activity across many species (42) and was hypothesized to influence age-related physical activity reductions in humans (43). Single nucleotide polymorphism (SNP) analysis suggested that the *DRD2* gene associated with physical activity levels in women (44) and that individuals with the CC homozygous variant in rs1800955 of the *DRD4* gene were more prone to sport-specific sensation seeking (45). Similarly, Wilkinson et al. (46) found associations between SNPs in two dopamine pathway genes, angiotensin I converting enzyme (*ACE*) and synaptosomal-associated protein 25 (*SNAP25*), and decreased likelihood for physical activity in youth.

However, whether alterations in the dopamine system are the result or driver of differences in voluntary physical activity is unknown. For example, previous reports show that voluntary wheel running is rewarding, and over time, able to alter behavior and affect the neuroplasticity of the mesolimbic reward pathway (34). Furthermore, endurance exercise training increased central dopamine concentrations up to 1.5-fold (47). Thus, physical activity, itself, could function in a feed-forward mechanism to further elevate physical activity.

## Obesity and Dopaminergic Dysregulation

**Figure 1:**
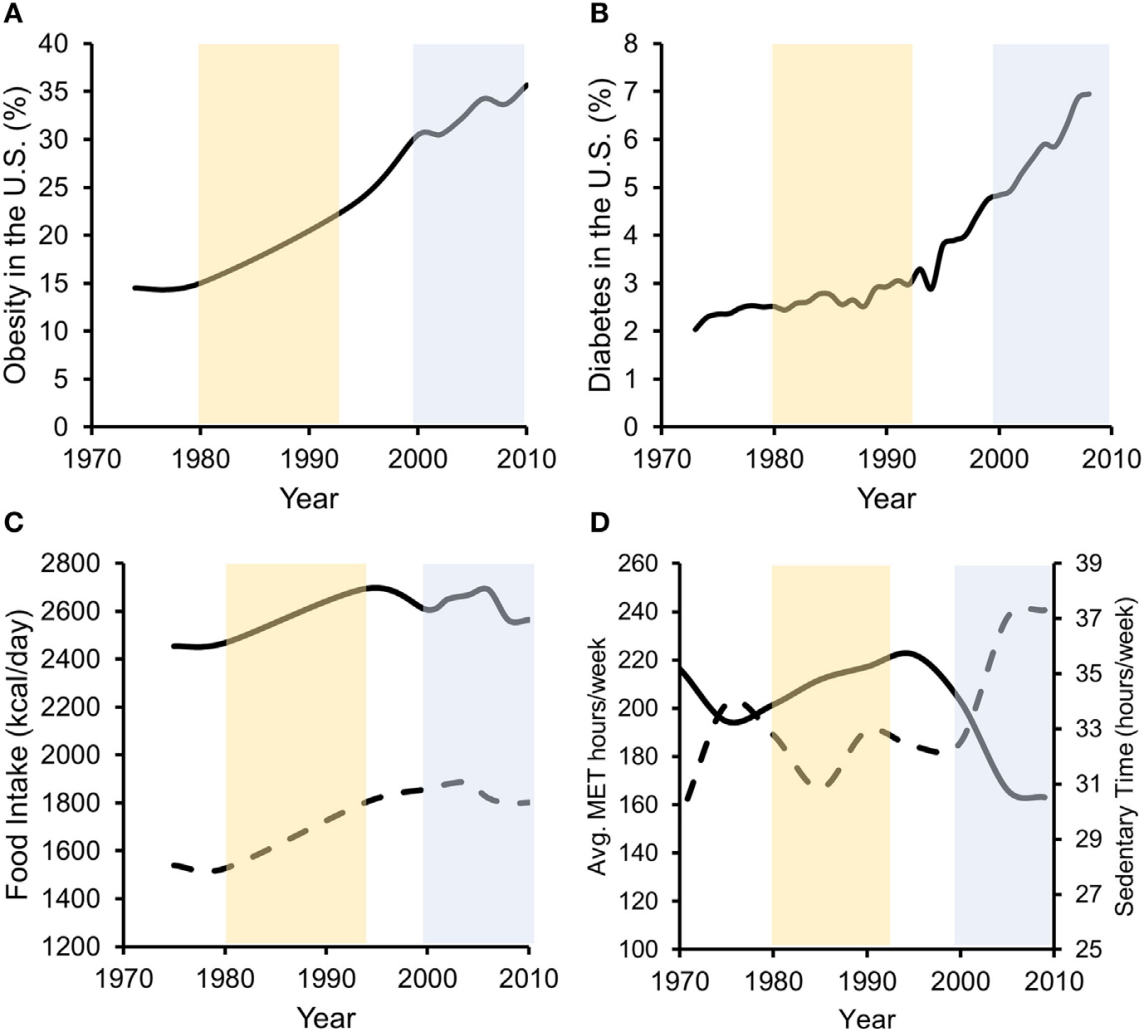
**Data suggest that both increases in energy intake and reductions in energy expenditure associate with increased obesity prevalence, while in later years, decreased energy expenditure more strongly associates with T2D prevalence**. Percentage of US adults with obesity **(A)** or diagnosed with type 2 diabetes **(B)** over the past ~40 years. **(C)** Unadjusted food intake for male (solid line) and female (dashed line) adults in the US during the same time frame. **(D)** Physical activity (solid line/left axis) [average metabolic equivalent (MET) hours per week] and physical inactivity (dashed line/right axis) (hours per week of sedentary time) performed by US adults. Obesity data redrawn from Ref. (48, 51), diabetes data from the CDC (52), food intake data from Ref. (53), and physical activity data from Ref. (54).

In the past three decades, obesity prevalence in the US has risen from below 20 to 36.5% (48). Additionally, physical inactivity levels and excessive food intake have increased over a similar period, directly contributing to increases in obesity and T2D (1) (Figure 1). Increases in unadjusted food intake from ~1980 to 1994 were associated with initial rapid increases in obesity, but not T2D, prevalence. Furthermore, beginning in ~1998 to 2000, physical activity levels rapidly dropped and sedentary time rapidly increased. This decrease in physical activity and increase in physical inactivity corresponded with increases in both obesity and T2D prevalence, despite food intake staying relatively constant during the same period. In our opinion, more recent increases in obesity are thus better associated with physical inactivity increases as caloric intakes were unchanged. Importantly, while declining physical activity levels contribute to obesity development, obesity contributed to reductions in physical activity in humans, even after controlling for baseline differences in physical activity (49). This interaction may promote the development of self-perpetuating vicious cycles whereby physical inactivity and obesity promote each other’s development (50).

The effects of obesity on the mesolimbic dopamine system are well studied, and hypotheses suggesting “reward dysfunction” in obesity have developed given findings that obesity is associated with alterations in striatal dopamine signaling (55). For example, reduced dopamine function, particularly DRD2 signaling, is associated with obesity development in rodents (56–59) and humans (60–62). However, these studies associated hyperphagia with obesity development and did not assess physical activity. Similarly, using positron emission tomography (PET) Guo et al. (63) observed a negative relationship between D2-like receptor binding in the ventral striatum and body mass index (BMI), suggesting that BMI could influence rewarding and effort-based actions. Similar measurements associating D1-like receptor neuron activity with obesity in humans are lacking, although several animal studies found that *Drd1* mRNA is reduced up to ninefold in the NAc of obese rats (64, 65). High-fat diet consumption for 12 weeks decreased tonic dopamine and *Drd1* and *Drd2* mRNA expression ~50% in the NAc of mice (66). Interestingly, following a 4-week recovery from high-fat diet, NAc *Drd1* and *Drd2* mRNA expressions were normalized in female, but not male, mice (66). Similarly, PET studies in humans show that DRD2 binding is not recovered (67) or partially recovered (68) following Roux-en-Y gastric bypass surgery. Collectively, these data suggest that reductions in dopamine function accompanying obesity could persist following weight loss. This notion is consistent with findings that physical inactivity levels remained high in obese humans months after weight loss (69–71), raising the question whether “physical activity resistance” exists temporarily/permanently after weight loss.

Interestingly, animal studies also suggest that high-fat diet exposure, rather than weight gain, may be more predictive of changes in striatal dopamine signaling. Isocaloric high-fat diet feeding in rats resulted in ~40% lower DRD2 in the NAc (72). Furthermore, chronic *ad libitum* high-fat diet reduced dopamine turnover 3.5-fold in the NAc of rats, although similar reductions were observed following isocaloric high-fat diet (73). Additionally, animal studies suggest that longer-term high-fat diet exposure can suppress dopamine synthesis, release, or turnover, ultimately reducing motivated behaviors not limited to motivation for food, such as physical activity (74). Despite considerable variability in experimental outcomes, we conclude that decreased dopamine signaling, particularly decreased D2-type function, could be particularly relevant to obesity.

## Obesity and Physical Inactivity

Obesity is strongly associated with physical inactivity (75, 76). While sparsely studied, several studies suggest that diet-induced dopaminergic alterations accompanying obesity may promote physical inactivity. Friend et al. (77) noted that diet-induced obesity in mice reduced D2-type receptor binding in the striatum that associated with decreased voluntary physical activity. Furthermore, in the same study the deletion of the *Drd2* gene, specifically in inhibitory medium spiny neurons (iMSNs), decreased wheel revolutions compared to littermate controls, although these mice were surprisingly not more vulnerable to diet-induced weight gain (77). Finally, the restoration of iMSN signaling reversed deficits in wheel running (77). Collectively, these data support the notion that D2-type receptor dysregulation contributes to obesity-induced physical inactivity, but that physical inactivity may be a consequence, rather than effector, of obesity.

Similarly, comparisons between mice bred for excessive exercise or obesity revealed that NAc dopamine content was increased in high running compared to obese and control mice, while *Drd1, Drd2*, and adenylate cyclase 5 (*Adcy5*) mRNAs were downregulated 92, 80, and 91%, respectively, in obese compared to control mice (78). Nonetheless, the authors hypothesized that modifications in the dopaminergic system may contribute to the differences in voluntary exercise between the high-running and obese mice (78). Analysis of obesity-resistant, compared to obesity-prone, rats also suggested that reduced physical activity levels in obesity-prone rats may stem from decreased action of hypothalamic orexin on dopamine neurons in the striatum and substantia nigra (79, 80). Finally, lower striatal dopaminergic activity may have contributed to low wheel running activity in rats with low aerobic capacity, who also had greater body weight and metabolic disease risk (40).

A recent study found that decreased DRD2 signaling in the striatum influences obesity development *via* reductions in physical activity rather than increases in food intake. Using *Drd2* knockdown mice, Beeler et al. (81) observed that when presented with voluntary exercise in an enriched environment, *Drd2* knockdown mice were dramatically less active than wild-type mice. Importantly, in the same study reduced voluntary exercise by *Drd2* knockdown mice promoted an obese phenotype despite no differences in food intake (81). These intriguing observations not only suggest a direct link between reduced dopamine function and decreased physical activity, but that the decreases DRD2 signaling can contribute to obesity *via* reduced energy expenditure rather than the initiation of compulsive overeating. Furthermore, obesity-induced reduction in DRD2 signaling could initiate the following feedback mechanism to further amplify obesity and physical inactivity: obesity → ↓ DRD2 signaling → ↑ physical inactivity → ↑ obesity → futile cycle. On the contrary, separate experiments show that dietary restriction increased wheel running (82) and dopamine overflow and receptor expression in the NAc (83, 84), suggesting that obesity and dietary restriction may have opposing effects on dopamine signaling and, in turn, voluntary physical activity. However, future research is needed to dissect causal and consequential relationships between obesity, dopamine, and physical inactivity.

## Central Leptin Action and Physical Activity

Relationships between leptin and physical activity are well established. Central leptin resistance is a hallmark of obesity (85, 86), and leptin resistance in the VTA following diet-induced obesity has been noted previously (87). Normal leptin signaling in VTA dopaminergic neurons is well characterized, with a general consensus being that leptin receptor (LEPR) signaling inhibits dopamine activity (88–90). Correspondingly, associations between select *DRD2* and *LEPR* allelic gene variations have been associated with the development of severe obesity (91).

**Figure 2:**
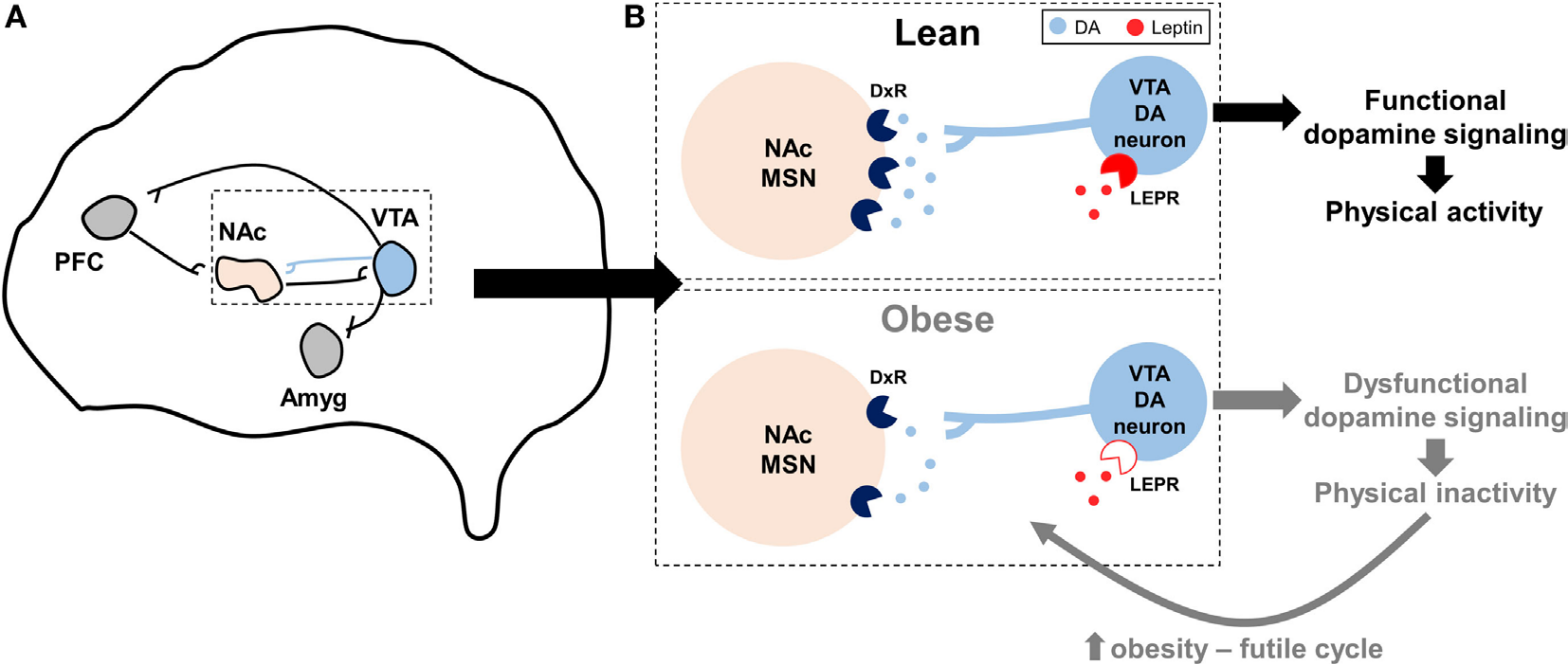
**Hypothesized model by which impaired dopaminergic signaling promotes physical inactivity in obesity**. **(A)** Summary of the reward circuitry in the brain; originally established by Robison and Nestler (95). The blue projection illustrates dopaminergic projections from the ventral tegmental area (VTA) that release dopamine (DA) onto post-synaptic neurons in the nucleus accumbens (NAc). **(B)** Expanded, but simplified, illustration of this dopaminergic VTA to NAc projection as it is hypothesized to relate to physical inactivity in lean and obese individuals. In obesity, dopamine receptor (DxR), particularly dopamine receptor 2, expression is decreased in NAc medium spiny neurons (MSNs). Similarly, mechanisms controlling DA production and release are reduced with obesity, leading to less DA in the synapse. Central leptin resistance in obesity [denoted by open leptin receptor (LEPR) symbol] may influence LEPR signaling in VTA DA neurons, in turn further diminishing downstream DA function. Collectively, these obesity-induced impairments in dopaminergic signaling may lead to exacerbated levels of physical inactivity, which may in turn lead to a futile cycle of increased obesity, dopaminergic dysregulation, and physical inactivity. Other abbreviations: Amyg, amygdala; PFC, prefrontal cortex.

Leptin suppressed the rewarding effects of wheel running in mice *via* activation of signal transducer and activator of transcription-3 (STAT3) signaling in VTA dopamine neurons, an effect which likely influenced dopamine overflow and function in the NAc and suggested that leptin may influence the motivational and rewarding effects of wheel running (92). Additional studies show that dopamine overflow in the NAc is reduced by leptin deficiency (88) and diet-induced obesity (57). In mice bred by Garland et al for high voluntary wheel running, which display dysfunctional dopaminergic profiles in the NAc as described above (36, 37), intraperitoneal leptin injection increased running by 17%, while control mice were unaffected (93). Paradoxically, in the same study high-fat feeding increased wheel running 20% in high-running mice, an effect speculated to be mediated by leptin (93). Intracerebroventricular injection of a recombinant adeno-associated virus (rAAV) overexpressing a mutant of leptin, which produces a protein that acts as a LEPR antagonist, decreased wheel running 25 and 40% in rats fed either a standard chow or high-fat diet, respectively, while rAAV overexpression of functional leptin increased wheel running ~2-fold Matheny et al. (94). However, changes in voluntary physical activity in the Matheny et al. study could be secondary to changes in adiposity following rAAV injection. Collectively, a hypothesis describing the interaction between obesity, dopamine, leptin, and physical inactivity is presented in Figure 2.

Further suggesting that leptin may impact the motivational and rewarding effects of running are observations that high serum leptin levels inversely correlated with low marathon run times after BMI adjustment (96), and with running performance (time and speed) in mice bred for high voluntary running (97). Leptin deficiency has also been shown to influence physical activity humans, whereas acute leptin increased locomotor activity in leptin-deficient patients during the fed state (98, 99). Similarly, leptin-deficient *ob/ob* mice increased wheel running 3.5-fold during the fed state following acute subcutaneous leptin injection, while no effect was observed in wild-type mice (100). Collectively, these studies highlight the important role of leptin as an effector of voluntary physical activity, potentially through alternations in dopamine signaling.

## Conclusion

Physical inactivity and obesity have reached pandemic levels (101). The abovementioned studies strongly suggest that dopaminergic function influences physical inactivity levels. Similarly, obesity-induced suppression of dopamine signaling may contribute to the high prevalence of physical inactivity observed in obese people. Additional understanding of mechanisms by which dopaminergic dysfunction contributes to obesity, physical inactivity, or their interactions may reveal novel approaches for increasing physically activity in obese populations.

## Author Contributions

GR and FB conceived the idea, wrote, and edited this manuscript.

## Conflict of Interest Statement

The authors declare that the research was conducted in the absence of any commercial or financial relationships that could be construed as a potential conflict of interest.
